# Dickkopf-1: A Promising Target for Cancer Immunotherapy

**DOI:** 10.3389/fimmu.2021.658097

**Published:** 2021-05-20

**Authors:** Hang Yin Chu, Zihao Chen, Luyao Wang, Zong-Kang Zhang, Xinhuan Tan, Shuangshuang Liu, Bao-Ting Zhang, Aiping Lu, Yuanyuan Yu, Ge Zhang

**Affiliations:** ^1^ Law Sau Fai Institute for Advancing Translational Medicine in Bone & Joint Diseases, School of Chinese Medicine, Hong Kong Baptist University, Hong Kong, China; ^2^ Guangdong-Hong Kong-Macao Greater Bay Area International Research Platform for Aptamer-based Translational Medicine and Drug Discovery, Hong Kong, China; ^3^ School of Chinese Medicine, The Chinese University of Hong Kong, Hong Kong, China; ^4^ Department of Microsurgery (II), Wendeng Hospital of Traditional Chinese Orthopedics and Traumatology of Shandong Province, Wendeng, China

**Keywords:** DKK1, cancer, Wnt signaling pathway, immunotherapy, immune surveillance

## Abstract

Clinical studies in a range of cancers have detected elevated levels of the Wnt antagonist Dickkopf-1 (DKK1) in the serum or tumors of patients, and this was frequently associated with a poor prognosis. Our analysis of DKK1 gene profile using data from TCGA also proves the high expression of DKK1 in 14 types of cancers. Numerous preclinical studies have demonstrated the cancer-promoting effects of DKK1 in both *in vitro* cell models and *in vivo* animal models. Furthermore, DKK1 showed the ability to modulate immune cell activities as well as the immunosuppressive cancer microenvironment. Expression level of DKK1 is positively correlated with infiltrating levels of myeloid-derived suppressor cells (MDSCs) in 20 types of cancers, while negatively associated with CD8^+^ T cells in 4 of these 20 cancer types. Emerging experimental evidence indicates that DKK1 has been involved in T cell differentiation and induction of cancer evasion of immune surveillance by accumulating MDSCs. Consequently, DKK1 has become a promising target for cancer immunotherapy, and the mechanisms of DKK1 affecting cancers and immune cells have received great attention. This review introduces the rapidly growing body of literature revealing the cancer-promoting and immune regulatory activities of DKK1. In addition, this review also predicts that by understanding the interaction between different domains of DKK1 through computational modeling and functional studies, the underlying functional mechanism of DKK1 could be further elucidated, thus facilitating the development of anti-DKK1 drugs with more promising efficacy in cancer immunotherapy.

## Introduction

### Characterization of Dickkopf-1 (DKK1)

DKK1 is an inhibitor of Wnt signaling pathway. Dysregulation of DKK1 has recently emerged as a potential biomarker of cancer progression and prognosis for several types of malignancies (1). Further research of DKK1 in oncology has garnered a lot of attention. The Dickkopf family (DKKs) are glycoproteins of 255-350 amino acids. The DKKs consist of four members in vertebrates (DKK1, 2, 3, 4), each containing two conserved cysteine-rich (Cys) domains involved in protein-protein interactions, which make the family unique. Apart from these, the family has no sequence similarity (2–4). Among the DKK family members, DKK1 is the best understood. DKK1 is a 266-amino acid protein with an approximate molecular mass of 26-kDa, and it possesses six secondary structures, which are two alpha helices and four β-sheets. It has the colipase-like fold in C-terminal Cys domain, which likely allows it to serve as an interactive surface (2, 5). DKK1 expression appears to be preferential in some adult tissues including bone, placenta, prostate, spleen, and colon (2, 6, 7). DKK1 has been shown to play an important role as a Wnt signaling pathway antagonist in several studies. For example, Dkk1 acts as an inducer in embryonic head formation and limb morphogenesis (8). Inhibition of Wnt signaling by Dkk1 promotes heart muscle formation in the anterior lateral mesoderm while repressing erythropoiesis (9). Moreover, since overexpression of DKK1 in osteoblasts causes osteopenia, it is also regarded as a negative regulator of normal bone homeostasis (10).

### DKK1 Inhibition in Wnt Signaling Pathways

DKK1 was originally identified in Xenopus as an inhibitor of β-catenin-dependent Wnt signaling and later its human homologue was also proved to have the same function (2, 3). The mechanism of DKK1 blocking the Wnt signaling pathway is demonstrated by multiple groups. To initiate the β-catenin-dependent Wnt signaling, Wnt needs to bind to Frizzled (FZD) Wnt receptors and low-density lipoprotein receptor-related proteins 5 and 6 (LRP5/6). DKK1, for antagonizing canonical Wnt/β-catenin signaling, does not directly interact with Wnt or FZD but forms complexes with LRP6 on the cell surface (11, 12). With the role of high affinity antagonistic ligand for LRP6, DKK1 inhibits the interaction of Wnt, FZD and LRP6, finally resulting in β-catenin degradation and inactivation of β-catenin/T-cell specific factor (TCF) transcription complex as well as consequent downregulation of a set of downstream genes regulated by T cell factors (13, 14). Remarkably, the *DKK1* gene is proven to be one of these downstream genes targeted by *TCF*, which indicates that DKK1 might have a self-regulatory function when suppressing *TCF* gene expression through Wnt signaling antagonist, thus forming a novel Wnt signaling inhibition negative feedback loop (15). According to the binding structure of DKK1 and LRP6, DKK1 could induce LRP6 to achieve a compressed conformation so that to preclude LRP6 from binding to the other ligands (16). Due to the inhibitory function of DKK1 in β-catenin-dependent Wnt signaling, which was a frequently overactivated pathway in cancer, DKK1 was originally characterized as a tumor suppressor (17, 18). It was revealed that DKK1 expression was decreased in gastrointestinal tumors and the gene was frequently silenced (19). Additional studies indicated DKK1 could suppress tumor growth and proliferation by inducing apoptosis of cancer cells (20, 21). However, since Wnt signaling has been recognized as one of the most significant oncogenic pathways correlated with immune evasion, this controversy is now being gradually overturned (22, 23). More importantly, there is growing evidence that DKK1 plays an essential role in cancer progression. For example, a recent study has demonstrated that DKK1expression helps cancer cells possess stem cell-like properties by preventing activation of β-catenin, thereby avoiding elimination from natural killer (NK) cells (24).

Despite findings supporting the regulation function of DKK1 in β-catenin-dependent Wnt signaling, DKK1 exhibited more complex intervention as it has also been linked to the activation of β-catenin-independent Wnt signaling. In osteosarcoma, the planar cell polarity-like (PCP) Wnt signaling pathway, which consisted of Ras homolog family member A (RhoA), mitogen-activated protein kinase kinase-4 (MKK4) and Jun N-terminal Kinase (JNK), was activated when DKK1 inhibited Wnt signaling, resulting in transcriptional activation of cancer stem cell marker aldehyde-dehydrogenase-1 (ALDH1) and promotion of tumor progression (25). The mechanism of DKK1 activation of β-catenin-independent Wnt signaling remains unclear, although the common opinion is that DKK1 is capable of inducing signal transmission from the β-catenin-dependent pathway to β-catenin-independent pathways. The effect of DKK1 on cell function is therefore hypothesized to include the modulation of both β-catenin-dependent and independent Wnt pathways, thus further enhancing the difficulty of regulating the Wnt signaling mechanism.

The identification of the novel DKK1 receptor cytoskeleton-associated protein 4 (CKAP4) provides a new perspective other than Wnt signaling pathway. Further characterization indicates that CKAP4 interacted with phosphatidylinositol 3-kinase (PI3K) and DKK1, leads to the activation of Akt signaling and increases the proliferation of the cancer cells (26, 27). These data suggest a scenario in which DKK1 can signal through a Wnt receptor-independent pathway to promote tumor growth. More evidence is needed to support this hypothesis.

## Over-Expression of DKK1 in Cancers

### Clinically Elevated Levels of DKK1 in the Serum and Tissue Samples of Cancer Patients

In recent clinical studies, dysregulation of the DKK1 was highlighted in various cancers including angiogenesis, metastasis, and other advanced invasion, and this was usually associated with poor prognosis (1). From the meta-analysis evaluating the relationship of DKK1 overexpression and prognosis of patients with gastric cancer, DKK1 was defined to be a prognostic marker with its association to vascular and lymphatic invasion, distant metastasis and low overall survival (28). According to other findings, DKK1 overexpression was also observed in breast cancer, ovarian serous carcinoma, lung and esophageal carcinoma (29–31). More importantly, elevation of both serum DKK1 levels detected by enzyme-linked immunosorbent assay (ELISA) and DKK1 protein levels assessed by immunohistochemistry has been found in different kinds of cancers including breast cancer, pancreatic ductal adenocarcinoma, esophageal squamous cell carcinoma, and non–small cell lung cancer, and this was in accordance with the cancer metastases or poor prognosis (31–33). The other studies also indicated the role of DKK1 as a novel serologic and prognostic biomarker due to its increased serum concentrations in various cancers, including gynecological cancer (34), prostate cancer (35), hepatocellular carcinoma (36), bladder cancer (37), lung cancer (38), multiple myeloma (39), and osteosarcoma (40).These data implied that DKK1 could be a novel diagnostic and prognostic biomarker and a promising therapeutic target for many cancers.

### Gene Expression Profiles From the Cancer Genome Atlas (TCGA)

**Figure 1:**
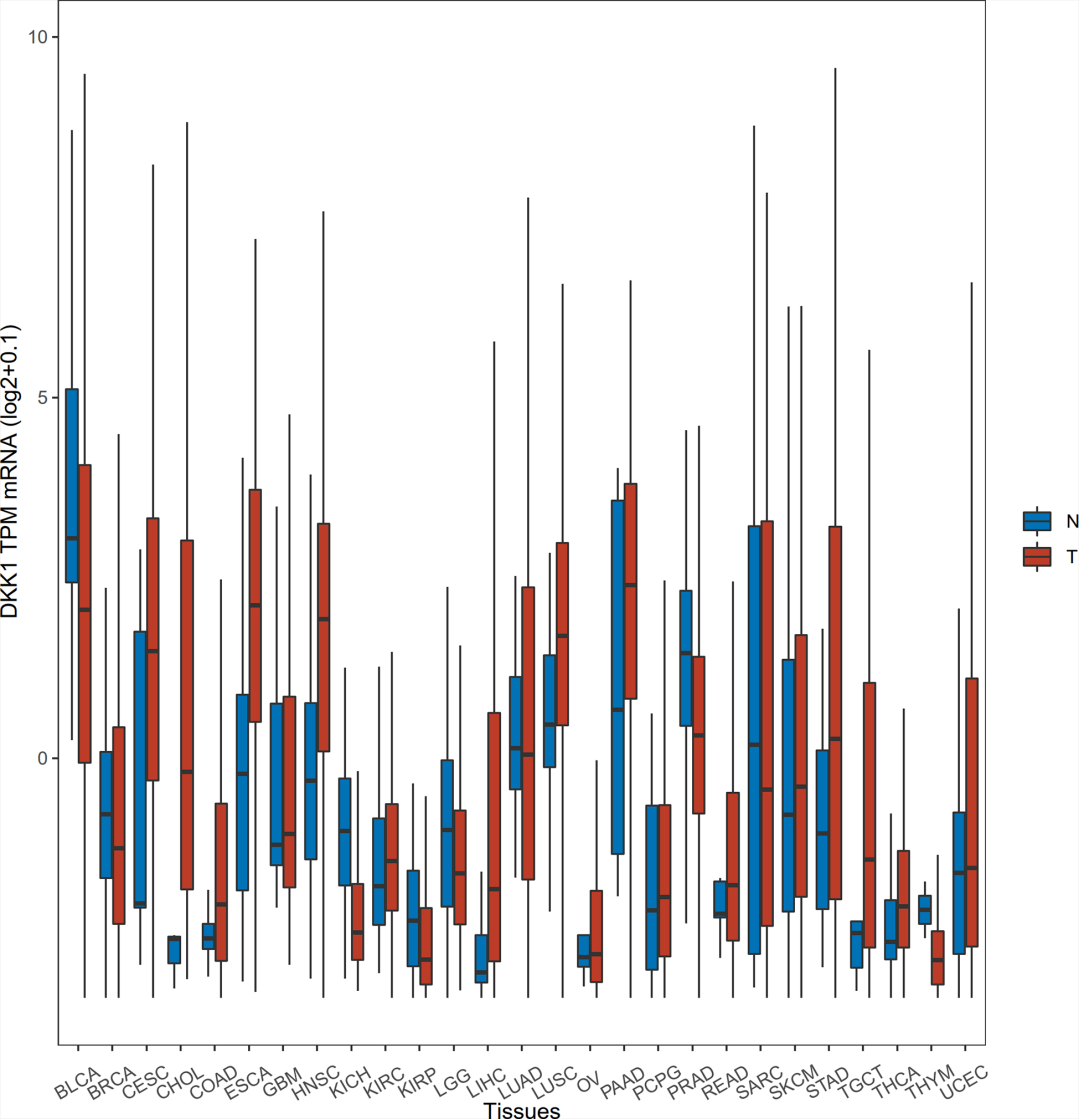
Differential expression of DKK1 in different disease states. The horizontal bars from bottom to top within the box represent the 25th percentiles, median, and 75th percentiles of the normalized DKK1 TPM mRNA. The upper and lower ends of vertical line correspond to the maximum and minimum of the normalized DKK1 TPM mRNA.

To acquire the latest *DKK1* gene expression profiles in a more comprehensive manner, we conducted statistical analysis with samples from TCGA data portal. The level-3 RNA-sequencing expression data (Fragments Per Kilobase Million (FPKM)) was retrieved by ‘TCGAbiolinks’ R packages (41). In this study, a total of 9677 tumor samples and 916 normal samples were downloaded. Subsequently, FKPM was transformed to Transcripts Per Kilobase Million (TPM) values using the formula TPMi = FPKMi/sum (FPKMj) x 10^6^. The FKPM value was normalized by (log2+0.1). We specifically selected the mean values of normalized *DKK1* TPM mRNA to do the *Student’s t-test* to verify the differential expression pattern in various tumor types (**Table 1** and **Figure 1**). The results demonstrated that *DKK1* mRNA was overexpressed in a wide range of tumors (14 out of 27 types analyzed), such as esophageal, colon, stomach, and uterine corpus endometrial carcinoma, providing diverse research directions about the roles of DKK1 in tumors. Consistent with other bioinformatic analysis, a significant overexpression of DKK1 was revealed in head and neck squamous cell carcinoma (HNSC), pancreatic adenocarcinoma (PAAD), and lung squamous cell carcinoma (LUSC), with DKK1 overexpression being associated with shorter disease-free survival (DFS) (42). These results implied that DKK1 could be an advantageous serologic biomarker indicative of tumorigenesis and poor prognosis.

**Table 1:** Differentially expressed analysis of DKK1 between Tumor and Normal samples in Pan-cancer Texts in red indicate carcinomas with significant higher DKK1 expression level compared to normal sample (P<0.05).

Abbreviation	Name	Normal Sample	Tumor Sample	P.Value
**BLCA**	Bladder Carcinoma	19	414	0.004
**BRCA**	Breast Invasive Carcinoma	120	1102	0.256
**CESC**	Cervical Squamous Cell Carcinoma and Endocervical Adenocarcinoma	5	304	0.159
**CHOL**	Cholangiocarcinoma	9	36	0.001
**COAD**	Colon Adenocarcinoma	43	478	0.001
**ESCA**	Esophageal Carcinoma	12	161	0.009
**GBM**	Glioblastoma	18	156	0.822
**HNSC**	Head-Neck Squamous Cell Carcinoma	46	500	0.001
**KICH**	Kidney Chromophobe	24	65	0.001
**KIRC**	Kidney Renal Clear Cell Carcinoma	73	538	0.305
**KIRP**	Kidney Renal Papillary Cell Carcinoma	33	288	0.247
**LGG**	Low Grade Glioma	18	511	0.309
**LIHC**	Liver Hepatocellular Carcinoma	53	371	0.001
**LUAD**	Lung Adenocarcinoma	61	533	0.273
**LUSC**	Lung Squamous Cell Carcinoma	49	502	0.001
**OV**	Ovarian Cancer	5	374	0.858
**PAAD**	Pancreatic Adenocarcinoma	5	177	0.387
**PCPG**	Pheochromocytoma and Paraganglioma	8	178	0.635
**PRAD**	Prostate Adenocarcinoma	53	498	0.001
**READ**	Rectum Adenocarcinoma	11	166	0.040
**SARC**	Sarcoma	6	259	0.810
**SKCM**	Skin Cutaneous Melanoma	103	369	0.389
**STAD**	Stomach Adenocarcinoma	32	375	0.001
**TGCT**	Testicular Germ Cell Tumors	6	150	0.001
**THCA**	Thyroid Carcinoma	66	502	0.001
**THYM**	Thymoma	2	119	0.424
**UCEC**	Uterine Corpus Endometrial Carcinoma	36	551	0.004

### Correlation Between Expression of DKK1 and β-catenin in Cancer Tissues

Along with the high DKK1 level, the β-catenin accumulation was found to be elevated in tumor tissues by immunohistochemistry (IHC) staining and this was linked to poor clinical outcome, which could be observed in chondrosarcoma, triple-negative breast cancer, hepatocellular carcinoma, and human hilar cholangiocarcinoma (43–46). We calculated the correlation between *DKK1* and *CTNNB1* (the gene that encodes β-catenin) by analyzing the mRNA levels in 10593 samples from the TCGA dataset. A positive correlation (correlation value: 0.13; *P < 0.0001*; Pearson’s correlation coefficient) was found between the two genes. The coexistence of DKK1 and β-catenin could be elucidated by a negative feedback mechanism. Since DKK1 has been identified as a target gene of activated β-catenin-dependent signaling, the abnormal activation of Wnt/β-catenin induced by mutation of other signaling components may promote β-catenin accumulation and thus provoke the elevation of DKK1 expression (15). The self-regulatory function of secreted DKK1 blocking its own transcription also supported this statement (47). However, a significant negative correlation was observable between β-catenin and DKK1 by IHC staining in epidermal neoplasms (48). This inconsistent result can be attributed to the variance in studies’ characteristics such as sample size, cancer staging, and location of tumor lesions, while it also suggests that the mechanism of DKK1 affecting cancer cells can be varied depending on cancers. Despite the positive correlation between *DKK1* and *CTNNB1* mRNA levels and protein levels, positive tissue microarray data of the two proteins did not match completely from hepatocellular carcinomas patients, suggesting that DKK1 expression could be mediated by other molecules, for instance, progesterone and transforming growth factor-β1 (TGFβ1) (45, 49, 50). Collectively, DKK1 is a potential predictor for the prognosis of a wide range of human cancers, along with a positive correlation with β-catenin expression.

## The Role of DKK1 in Modulating Cancer Cells

### Cancer-Promoting Effects of DKK1 in Cancer Models

Beyond the potential to be a diagnostic indicator, DKK1 has been viewed as a promising target for anti-cancer therapy due to its cancer-promoting activity existing in preclinical cancer models (**Table 2**). In these *in vitro* models, DKK1 could contribute to tumor progression by promoting migration, invasion, proliferation, preventing apoptosis and enhancing cancer stem cell-like properties. In addition, DKK1 had tumor-promoting activity in *in vivo* animal models through its effects on tumor growth, metastasis, and angiogenesis.

**Table 2:** The effects of DKK1 in cancer model and immune response.

Types of cancers	Cancer-promoting activities	Reference
**Cell Culture Model**		
**Breast**	DKK1 enhanced ECFC proliferation while DKK1 silencing suppressed their angiogenic potential.	(51)
	DKK1was highly expressed by human breast cancer cell lines with osteolytic bone features.	(52)
**Intrahepatic Cholangiocarcinoma (ICC)**	DKK1 enhanced tumor cell invasion and promoted lymph node metastasis through the induction of MMP9 and VEGF-C.	(53)
**Human hilar cholangiocarcinoma**	Depletion of DKK1 led to repression of cell proliferation and migration in part through the β-catenin/MMP-7 signaling pathway.	(46)
**Hepatocellular carcinoma**	DKK1 promoted HCC cell migration and invasion partly by regulating β-catenin/MMP7 signaling axis.	(54)
	Deletion of DKK1 suppressed cell migration and invasion while its overexpression showed opposite effect.	(55)
**Multiple myeloma**	Anti-DKK1 antibody inhibited proliferation of MM cells in co-culture with osteoclasts.	(56)
	Anti-DKK1 antibody hindered MM cell growth by regulating the bone marrow microenvironment.	(57)
**Colorectal**	DKK1 located in the nucleus of human colorectal cancer cells involved in cancer-related target gene transcription.	(7)
**Small cell lung cancer**	Reduced expression of DKK1 induced inhibition of cancer cell proliferation, colony formation, migration and invasion.	(58)
**Non-small cell lung cancer**	DKK1 overexpression enhanced proliferation, invasion, migration and vascular invasion of cancer cells.	(59)
	Anti-DKK1 antibody suppressed cancer growth and invasive activity.	(60)
**Lung and pancreatic**	Knockdown of DKK1 in cancer cells inhibited cancer cell proliferation and migration.	(26)
**Osteosarcoma**	DKK1 expression promoted cancer cell expansion and increased tumor stress metabolic resistance for its upregulation of ALDH levels.	(25)
**Esophageal**	DKK1 knockdown inhibited cell proliferation, colony formation and stem cell-like characteristics including downregulation of ALDH1A1 and CK18 expression.	(61)
***In Vivo* Animal Model**		
**Breast**	DKK1 strongly enhanced the vascularization of Matrigel plugs and tumor growth.	(51)
	Levels of Dkk-1 dramatically increased in bone marrow of inoculated mice.	(52)
	DKK1 showed dichotomous role in metastasis organotropism as it inhibited lung metastasis and promoted bone metastasis at the same time.	(62)
**Breast and lung**	DKK1 promoted malignant cell proliferation by facilitating LCC cells to enter quiescence and thus achieve immune surveillance.	(24)
**Human hilar cholangiocarcinoma**	DKK1 expression was positively correlated with tumor volume as well as MMP-7 expression.	(46)
**Multiple myeloma**	Anti-DKK1 antibody resulted in diminished tumor growth and prevented bone loss.	(56)
	Humanized anti-DKK1 antibody demonstrated significant anti-MM effect.	(57)
	A neutralizing DKK1 antibody reduced primary myeloma burden and increased bone formation.	(63)
**Small cell lung cancer**	Interfering with DKK1 helped prevent bone metastasis.	(58)
**Non-small cell lung cancer**	Silencing of DKK1 reduced tumor growth rate, tumor volume, and vasculogenic-related protein expression.	(59)
	Anti-DKK1 antibody inhibited tumor growth.	(60)
**Lung and pancreatic cancer**	DKK1 knockdown suppressed tumor formation	(26)
**Osteosarcoma**	DKK1 expression led to increased tumor formation and deterioration.	(25)
	DKK1 responded to the treatment of anti-DKK1 antibody BHQ880 with decreased tumor growth rate and metastasis.	(64)
**Hepatocellular carcinoma**	DKK1 induced increased lung metastasis rate.	(56)
**Prostate cancer**	DKK1 increased tumor burden as well as bone metastasis.	(65)
**Types of immune cells**	**Regulatory activities**	**Reference**
**MDSCs**	DKK1 contributed to MDSC accumulation and tumor progression *in vivo* by reducing β-catenin levels.	(24)
**T cells**	The increased circulating amounts of DKK1 polarized T cells to Th2 cells, which was mediated by the kinases p38 MAPK and SGK-1, leading to Th2 cell cytokine production.	(66)
**NK cells and CD8^+^ T cells**	Overexpression of DKK1 could modulate the anti-tumor immune populations within the tumor mice.	(67)
**DCs and CD8^+^ T cells**	Anti-tumor efficacy of anti-DKK1 DNA vaccine in mice model required the function of CD8^+^CD11c^+^ dendritic cells (DCs) and CD8^+^ T cells.	(68)
**MDSCs and CD45^+^ cells**	Antibody DKN-01 induced reduction in MDSCs and upregulation in CD45^+^ cells within the tumor microenvironment.	(69)
**CD4+ and CD8+ T cells**	DKK1 vaccine elicited strong CD4+ and CD8+ immune responses toward tumors.	(70, 71)

Emerging evidence has improved our understanding of how DKK1 could promote tumor growth and metastasis through the modulation of signaling pathways in cancer cells. For example, a recent study has demonstrated that DKK1 promoted metastasis in an *in vivo* model attributed to its function of inhibition of β-catenin-dependent Wnt signaling (24). Latency competent cancer (LCC) cells showed stem cell-like characteristics and expressed Sry-box transcription factor 2 (SOX2) and Sry-box transcription factor 9 (SOX9) transcription factors, which were critical for their survival in host organs under immune surveillance and for metastatic outgrowth under permitted conditions. In the observation of LCC cells isolated latency from early-stage human lung and breast carcinoma cell lines, these cells were found to enter quiescence by self-imposing a slow-cycling state to avoid immune surveillance. Impeding DKK1 expression would re-sensitize these LCC cells to β-catenin-dependent Wnt signaling and upregulate the expression of activating ligands for NK cells, leading to NK cell-mediated clearance of the LCC cells and reduced metastasis. The result implied that the reactivation of β-catenin-dependent Wnt signaling, in a way that suppressed DKK1, could contribute to the elimination of LCC cells and thereby prevent tumor metastasis. Corresponding to the impact on stem cell-like LCC cells, DKK1 could favor the formation of an undifferentiated phenotype *via* inhibiting β-catenin-dependent Wnt signaling. For example, DKK1 antibody restored β-catenin-dependent Wnt signaling and promoted phenotype differentiation in osteosarcoma, leading to less distant metastases (64). Cancer stem cells have been linked to cancer metastases and drug resistance due to their early existence during tumorigenesis and reversible states from active to quiescence (72). The function of DKK1 on cancer cells with stem/progenitor cell features suggests that DKK1 may be involved in cancer stem cells-related phenotype development and cancer cell dissemination. Some concerns have arisen since re-activation of phenotype cells may also induce proliferation of the cells themselves and, in principle, increase tumor growth. How to enhance tumor immune surveillance without promoting tumor growth is an important issue to be addressed.

Another recent study demonstrated the ability of DKK1 to facilitate invasive cell behaviors by increasing mesenchymal characteristics in receptive cells. DKK1 was proven to be involved in disturbing cell cohesion and altering cell polarity, resulting in unsuccessful cell-cell adhesion and coordinated progression. This cell interaction control was independent of β-catenin transcriptional output and Wnt/PCP signaling (73). Additionally, the Janus-faced molecules feature of DKK1 in organotrophic metastasis was deciphered in another study (62). In breast cancer, tumor-secreted DKK1 suppressed lung metastasis by downregulating PTGS2-induced macrophage and neutrophil recruitment by antagonizing non-canonical SOX2 and SOX9 signaling. In the lungs, DKK1 also reduced the latent transforming growth factor-beta binding protein-1 (LTBP1)-mediated transforming growth factor-beta (TGF-β) secretion of cancer cells by inhibiting Wnt/Ca^2+^-calmodulin-dependent protein kinase II) (CaMKII)-Nuclear factor-κb (NF-κB) signaling. In contrast, DKK1 promoted breast-to-bone metastasis by regulating canonical Wnt signaling of osteoblasts. Given that the TGF-β signaling pathway was also of great importance for osteolytic metastasis (74), it was intriguing to observe that suppression of LTBP1 and TGF-β under DKK1 overexpression did not interfere with bone metastasis. This obvious inconsistency could be interpreted by the rich reservoir of TGF-β in bone matrix and thus the reduced TGF-β of DKK1-expressing tumor cells may be easily compensated. These results suggest that the combinatory targeting strategy of non-canonical Wnt signaling that included JNK and TGF instead of canonical Wnt signaling may be more beneficial for metastatic diseases therapy. In view of this, the therapeutics approach against DKK1 should be examined regarding the dichotomous function of DKK1 in cancer metastasis organotropism.

## The Role of DKK1 in Modulating the Immune Cells

### Correlation Between DKK1 Expression and Tumor-Infiltrating Lymphocytes

**Figure 2:**
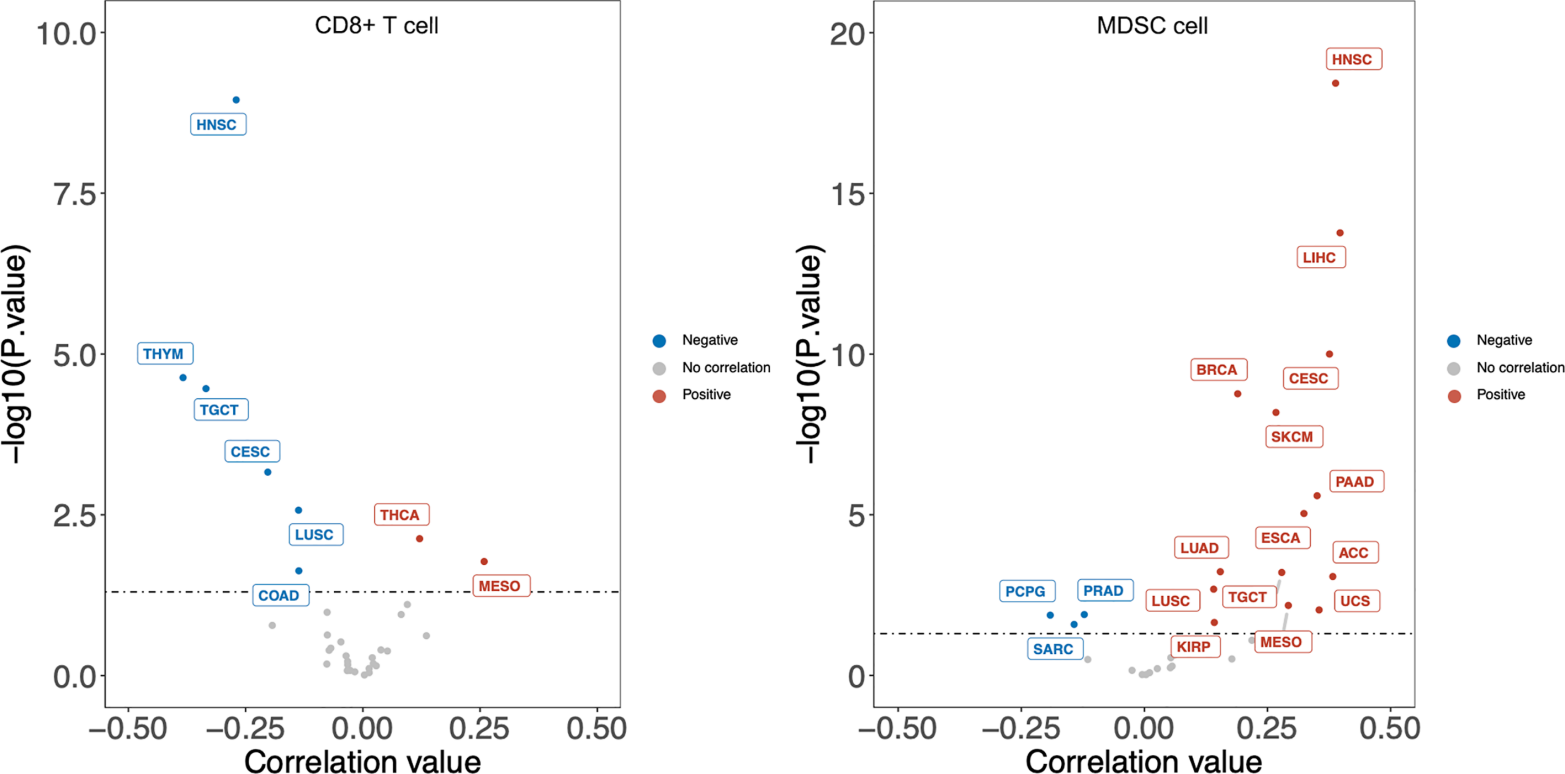
*DKK1* expression level has significant negative correlation with infiltrating levels of CD8^+^ T cells and positive correlation with MDSC cells in various cancers. According to the results, the expression level of *DKK1* was positively correlated with infiltrating levels of MDSCs in 20 types of cancers, and negatively correlated with levels of CD8+ T cells in 6 types of cancers. Cancers that fit these two relations include HNSC, TGCT, CESC, and LUSC. HNSC, Head-Neck Squamous Cell Carcinoma; THYM, Thymoma; TGCT, Testicular Germ Cell Tumors; CESC, Cervical Squamous Cell Carcinoma and Endocervical Adenocarcinoma; LUSC, Lung Squamous Cell Carcinoma; COAD, Colon Adenocarcinoma; THCA, Thyroid carcinoma; MESO, Mesothelioma; LIHC, Liver Hepatocellular Carcinoma; BRCA, Breast Invasive Carcinoma; SKCM, Skin Cutaneous Melanoma; PAAD, Pancreatic Adenocarcinoma; ESCA, Esophageal Carcinoma; LUAD, Lung Adenocarcinoma; ACC, Adrenocortical Carcinoma; UCS, Uterine Carcinosarcoma; KIRP, Kidney Renal Papillary Cell Carcinoma; PRAD, Prostate Adenocarcinoma; PCPG, Pheochromocytoma and Paraganglioma; SARC, Sarcoma.

Previous analyses implied tumor-infiltrating lymphocytes could be independent predictors of sentinel lymph node status and survival in cancer patients (75). To have a clear understanding of the relation between DKK1 and immune infiltrates across a spectrum of cancer types, we employed the TIMER2.0 as a comprehensive resource for systematic analysis (http://timer.comp-genomics.org/) (76). TIMER applies a previously published statistical approach called deconvolution that uses gene expression profiles to produce an inference in the number of tumor-infiltrating immune cells. TCGA provides the TIMER database 10,897 samples across 40 cancer types, which are applied for the approximation of immune infiltrates. We inquired on the mRNA expression of *DKK1* in different types of cancers and its correlation with the abundance of immune infiltrates. From the results, the expression level of DKK1 was significantly positively correlated with infiltrating levels of MDSCs in 20 types of cancers, suggesting DKK1 is involved in MDSCs modulation in a variety of cancers (**Figure 2**). More importantly, there was a negative correlation between levels of *DKK1* and CD8^+^ T cells in the corresponding carcinomas, including HNSC, TGCT, CESC, and LUSC. MDSCs were defined as the heterogeneous population of immature myeloid cells recruited by tumors, which could induce CD8^+^ T cell tolerance in tumor-bearing hosts despite seemingly adequate infiltration by CD8^+^ T cells and interferon-responsive tumor cells (77). Results of the analysis indicated DKK1 may induce MDSC accumulation and T cell dispersal during tumor progression.

### Immunoregulatory Effects of DKK1

Activation of the Wnt signaling pathway directly regulated the function of immune cells including dendritic cells, macrophages, myeloid-derived suppressor cells and T lymphocytes (78). The confirmed Wnt signaling inhibitor DKK1 was consequently assumed to intervene in immune cell activities. Recently, DKK1 was proposed as an indicator of inflammatory responses for its elevated levels in patients with infections or hematological disorders (79, 80). In the allergen challenge model, DKK1 enhance type 2 inflammation response and T helper 2 (Th2) cell polarization *via* serum glucocorticoid kinase (SGK)-1 and p38 mitogen-activated protein kinases (MAPK) in CD4^+^ T cells, while reducing T helper 1 (Th1) cytokine production (66). For instance, DKK1 suppressed interferon-γ (IFn-γ) from CD4^+^ T cells while elevating the secretion of Th2 cytokine including interleukin (IL)-4, IL-5, IL-10, and IL-13. The same preferential shift of Th1 to Th2 cells has also been found in the carcinogenesis process (81). Interestingly, inflammation driven by tumour-specific Th1 call is currently believed to prevent cancers but Th2 subset seems to have an opposite function (82). Th1-mediated interferon-γ (IFn-γ) is proved to be necessary for facilitating macrophages to kill cancer cells and up-regulating MHC molecules on cancer cells leading to enhance T cell recognition (83, 84). On the other hand, the Th2-type cytokine pattern is more preferential in the malignant tumor and participated in cancer progression and immune evasion (85–87). In addition, Chae’s work also revealed that platelets are the source of circulating DKK1 (66). Preclinical and clinical studies shows that platelets have a crucial role in tumorigenesis, metastasis, and cancer evasion of immune surveillance (88, 89). Based on these studies, platelet-derived DKK1 may be a pro-tumorigenic factor due to its various crosstalk with immune cells. Besides involving in modulation of T helper cell development, DKK1 was also proven to be a key player in tumor microenvironment through MDSCs manipulation. D’Amico et al. found that DKK1 was highly expressed in circulation and bone environments in syngeneic murine tumor models such as melanoma and Lewis lung carcinoma models, and the dominant source of DKK1 could be specific to bone-resident osteoblasts (90, 91). In this study, antibody-mediated neutralization of DKK1 resulted in a marked reduction of MDSCs expansion and tumor progression. The mechanism could be illustrated by the inhibition of β-catenin by DKK1 leading to accumulation of MDSCs and increasing their ability to suppress T cell activation and proliferation. Supporting this potential mechanism, Capietto et al. previously showed that DKK1-drived downregulation in β-catenin drives MDSC accumulation in the bone marrow, spleen, and at primary tumor sites, and controls their immune suppressive effects (91).

A recent study reveals that overexpression of DKK1 could modulate anti-tumor immune populations within the tumor microenvironment by decreasing CD45^+^ leukocyte infiltration and reducing NK and CD8^+^ T cells (67). On the other hand, a novel anti-tumor DNA vaccine designed by Guo’s team stressed the significance of the role of CD8^+^ cells in the effect of DKK1 on tumor immunosuppression (68). The vaccine targeting PD-L1 and DKK1 showed efficacy in the mice model of multiple myeloma, which required the function of CD8^+^CD11c^+^ dendritic cells and CD8^+^ T cells. To summarize, DKK1 had immunoregulatory effects, including promoting Th2 cells response, reducing the functionality of T cells through MDSC modulation and suppressing the proliferation of CD8^+^ T cells and NK cells, thus contributing to inflammatory response and cancer immune evasion. Although there has been a more detailed and clear description of the participation of DKK1 in immune response due to recent research, an investigation of how DKK1 manipulates different immune responses to exert anti-tumor effects is necessary. Active Wnt signaling pathway plays an essential role in T cell and B cell development, for example, mice with deficient for the Wnt-responsive transcription factors TCF1 and Lymphoid enhancer-binding factor 1 (LEF1) showed defects in T cell and B cell development (92) (93). Nonetheless, how the Wnt antagonist DKK1 could intervene in these cell processes is still poorly understood. More direct evidence is required to elucidate the mechanism of how DKK1 modulates immune cells for immunosuppression.

### Potential Role of DKK1 in Regulating Therapeutic Response of Immune Checkpoint Inhibitors

Some nonclinical studies for developing anti-DKK1 drugs have provided clues to reveal the pivotal role of DKK1 in regulating therapeutic response of immune checkpoint inhibitors. For example, the activity of the anti-DKK1 antibody DKN-01 was dependent on a functional immune system in the murine cancer model. Furthermore, DKN-01 induced reduction in MDSCs and upregulation in CD45^+^ cells in the tumor microenvironment (69). This evidence suggested DKK1 would participate in anti-tumor immunomodulation. Local immunosuppressive factors within the tumor microenvironment, including MDSCs, were regarded as one of the mechanisms of primary resistance of PD-1/PD-L1 therapy. MDSCs were defined as the heterogeneous population of immature myeloid cells recruited by tumors, which were able to impair response to a PD-1/PD-L1 blockade despite seemingly adequate infiltration by CD8^+^ T cells and interferon-responsive tumor cells (77). Possibly, the DKK1 antibody could act on MDSCs to exploit the supportive tumor microenvironment by preventing MDSC accumulation and compromising their inhibitory effect on immune cells. In addition, some innovative inhibitory strategies against DKK1 have been reported. Park et al. generated a cyclic oligopeptide that could inhibit Wnt signaling pathway and reduce tumor burden *in vivo* (94). A fusion DNA vaccine composed of DKK1 and human HSP70 developed by Liu et al. and a DKK1 DNA vaccine produced by Qian et al. also consistently demonstrated therapeutic efficacies on murine multiple myeloma (70, 71). In the latter study, injection of the DKK1 vaccine not only repressed tumor growth, but also enhanced CD4^+^ and CD8^+^ T cell response toward cancer cells. Further understanding of DKK1 in immunotherapy requires more experimental evidence, which may prompt the use of DKK1 antibody in combination with other existing immunotherapeutic drugs to achieve better efficacy.

## Development of Anti-DKK1 Therapy

### Anti-DKK1 Antibody in Clinical Trials

DKK1’s suppressive effect on the immune system made it a promising target for immunotherapy. Recently, the anti-DKK1 neutralizing antibody DKN-01, which is developed by Leap Therapeutics, are being evaluated in phase I or phase II trials of advanced gastroesophageal junction and gastric cancer (GEJ/GC) and gynecologic cancers, in the form of both monotherapy and combination therapy with paclitaxel or pembrolizumab. In the phase II trials of advanced gynecologic malignancies (relapsed or refractory epithelial endometrial cancer, epithelial ovarian cancer, or carcinosarcoma: NCT03395080), the overall response rate (ORR) of DKN-01 monotherapy was 7% while that of combination with paclitaxel was 4% (95). Of note, gynecologic cancer patients with Wnt signaling mutations were beneficial from DKN-1 treatment (96). The other ongoing clinical study of GEJ/GC demonstrated improved survival and objective response outcomes especially in patients whose tumors expressed high levels of DKK1 (DKK1-high) (97). DKN-01 plus Keytruda (pembrolizumab) combination therapy was used to treat sixty-three patients in all different arms and dose. Around 84% patients had never undergone PD-1/PD-L1 therapy before, and ten were PD-1/PD-L1 treatment refractory. All patients in the study had been heavily pretreated, with one to five lines of therapy already administered. The highly encouraging results stated that DKN-01 plus Keytruda therapy resulted in a median progression-free survival (PFS) over 22 weeks and a median overall survival (OS) nearly 32 weeks, with a 50% ORR and 80% disease control rate (DCR) in patients with DKK1-high GEJ/GC who had not received prior anti-PD-1/PD-L1 therapy. Among the six GEJ/GC patients who were refractory to PD-1/PD-L1 therapy, three patients with high serum levels of DKK1 had the best response of stable disease, whereas the three patients with low serum levels of DKK1 had progressive disease (97). The results revealed that DKK1 inhibition therapy significantly improved the responsiveness of patients with high serum levels of DKK1 and suggested the strategy to achieve better efficacy using DKK1 inhibitor along with PD1/PDL-1 immune checkpoint treatment in patients who have DKK1-high tumors. Another DKK1 antibody BHQ880, developed by Novartis Pharmaceuticals, had completed phase 1B trials in multiple myeloma. Despite some observed clinical benefits, 96.4% patients (n=28) reported adverse events (AE) with one-third of these reported events suspected to be related to either the antibody or zoledronic acid. The suspected study drug related AEs include hypertension (n=2), increased serum creatinine (n=1) and thrombocytopenia (n=1). Further clinical studies are needed to evaluate these monoclonal antibodies’ efficacy and safety in the treatment of individual cancer. More significantly, given its unsatisfactory performance relative to combination therapy, how to enhance the therapeutic efficacy of DKK1 antibody monotherapy was an important topic, in which understanding the functional mechanism as well as the 3D structure of DKK1 could provide potential directions.

### Function of DKK1 Domains and Prediction of the 3D Structure of DKK1

**Figure 3:**
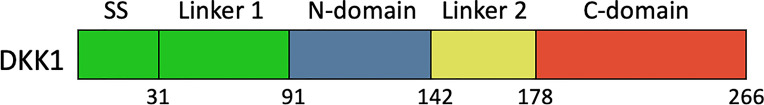
Schematic diagram of the primary structures of full length DKK1. SS, signal sequence; N-domain and C-domain, two conserved cysteine-rich domains; L1, N1, L2, C1, named domain construct.

DKK1 consisted of five domains, including signal sequence, Linker 1 (L1), the amino-terminal cysteine-rich domain (termed N), Linker 2 (L2), and the carboxyl terminal cysteine-rich domain (termed C) (2) (**Figure 3**). So far, several studies focused on the functional mechanism of the feature cysteine-rich N domain and C domain have been conducted. Crystal structure demonstrated that C domain and N domain bind to the four β-propeller and EGF-like domain repeats of LRP6 ectodomain, respectively (98). Because these domain repeats were capable of binding to several Wnt variants, a single DKK1 molecule may bind to all portions of the LRP6 ectodomain and thereby inhibit different Wnt pathways. The C domain was regarded as the critical binding site for Wnt signaling inhibition and the antibody DKN-01 could specifically recognize the C domain of DKK1 (100). More strikingly, a study on Wnt signaling in *Xenopus* embryos assessed distinct functional activities of these two functional domains. The C domain was shown to be necessary and sufficient for Wnt inhibition, while the N domain regulated this interaction by masking the ability of the C domain to synergize with LRP6 (101). On the other hand, the function of the N domain was more perplexing. It was defined as a signal domain that was responsible for the potent effects of DKK1 on heart-induction when synergizing with Wnt antagonists (102). CKAP4 was originally known as a protein that mainly localized to the endoplasmic reticulum and is now also defined as a novel DKK1 receptor. In the case of DKK1/CKAP4 signaling pathway, the N domain was found to be required for DKK1 binding to CKAP4 and thereby activated AKT by forming a complex between the leucine zipper domain of CKAP4 and the Src homology 3 domain of PI3K, resulting in cellular proliferation (26). This finding suggested that the N domain along with canonical Wnt inhibitor may play a crucial role in the numerous developmental and disease processes that involve DKK1. From this result, DKK1 may self-regulate its inhibitory effect on the Wnt signaling pathway. One possibility was that the N domain interacted with the C domain, thereby inhibiting its ability to synergize with LRP6. Furthermore, the post-translational modification of DKK1 protein may provide some useful hints. Ten disulfide bonds were detected in DKK1 molecule with five in the N domain and five in the C domain (102). Interesting, the disulfide topographic arrangement in the same domain is similar, while the overall pattern of all five disulfide bonds in these two domains is apparently different, suggesting that the two domains may have a different role in DKK1 function. There are nine reported glycosylation sites including the *O*-linked glycosylation at Ser61, Thr155, Ser163, Thr164, Ser169, Thr172, Thr173, Thr181, and *N*-linked glycosylation Asn256, which may interferes with the secretion of protein (103–105). Moreover, the L 1 and L2 domains may also be involved in controlling the protein conformation.

**Figure 4:**
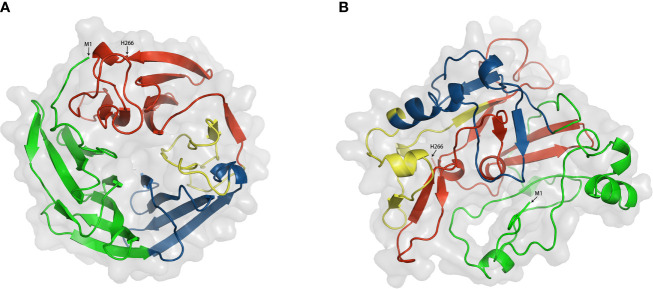
Two modeling structures of DKK1. **(A)** a circular conformation; **(B)** a closed conformation. Green. Signal peptide (1–31); Link1 domain (32–91); Blue, N domain (92–142); yellow, Link2 domain (143-178); Red, C domain (179-266). Interactions between two residues were defined when the atoms distance was < 4 Å, and strong interactions between two residues were defined when the atoms distance was < 3 Å.

For the purpose of further depicting the functional mechanism of DKK1, it is essential to explore the roles of different domains. However, no three-dimensional protein structure of full length DKK1 was reported at present. According to the existing reports mentioned, we hypothesized that DKK1 could form distinct conformations with different intermolecular interactions between C domain and N domain, resulting in differences in the intramolecular interactions with LRP6 and CKAP4, respectively. To have a better understanding of DKK1 structure, we predicted the 3D structure of full length DKK1 by homology modeling and calculate the inter-molecular interactions between domains within DKK1. ORION was employed for predicting protein 3D structure and this method relies on the descriptors called Protein Blocks (PB) to encode a structural alphabet defined by 16 local structural patterns that accurately describe local protein structures (58, 106). Interactions between two residues were defined when the atoms distance was < 4 Å, and strong interactions between two residues were defined when the atoms distance was < 3 Å. According to the modeling results, DKK1 mainly formed two different 3D structures (**Figure 4** and **Table 3**). One was a circular conformation with no interactive residues between C domain and N domain but considerable interactive residues between N domain and L2 (five pairs of strongly interactive residues), which could hinder the interaction between N domain and C domain. The other was a closed conformation with most interactions forming between N domain and C domain (11 pairs of strongly interactive residues). The interactive residues between N domain and L2 was less than that in the circular conformation (three pairs of strongly interactive residues). Since N domain was reported to diminish the binding of C domain to LRP6 (100), the closed conformation would be more desirable for impeding DKK1 from Wnt signal antagonizing. Moreover, these results suggested that the L2 domain of DKK1 may affect the 3D structure by competing with C domain for binding to N domain. Thus, intervening in the interaction between L2 and N domain may be a promising approach to facilitate the formation of the closed structure. Further functional studies are required to validate the results of the prediction and help to give a more insightful understanding of how the two domains of DKK1 interact structurally and thus provide insight into the development of more effective inhibitors. In addition, further exploration of the different configurations of these two regions and the interaction sites enable the intervention by specifically targeting inhibitors to achieve the ideal configuration of DKK1. This could provide a strong basis for developing advance DKK1 inhibitors with high anti-tumor efficacy.

**Table 3:** The inter-molecular interactions among domains within DKK1.

Domain	Structure 1		Structure 2	
	Residues pair	Distance (Å)	Residues pair	Distance (Å)
**Link 2 & N**	E-149 & C-114	3.2	L-174 & R-102	2.9
	I-150 & C-114	3.5	K-177 & R-102	3.0
	I-150 & L-112	3.1	N-177 & P-100	3.1
	E-151 & P-100	3.8	N-144 & S-140	2.6
	E-151 & L-112	3.0	/	/
	E-152 & L-112	3.0	/	/
	Y-168 & I-137	2.9	/	/
	H-162 & V-139	2.8	/	/
	R-170 & R-102	3.0	/	/
	L-174 & G-104	3.4	/	/
	L-174 & Y-132	3.6	/	/
	S-176 & C-133	3.2	/	/
**C & N**	/	/	Y-179 & A-98	3
	/	/	Y-179 & P-100	3
	/	/	H-180 & E-95	3.3
	/	/	V-188 & C-97	3.1
	/	/	S-193 & A-98	2.7
	/	/	R-224 & M-126	2.9
	/	/	S-228 & N-131	2.6
	/	/	L-231 & R-115	3.1
	/	/	E-232 & A-113	2.6
	/	/	E-232 & R-118	2.7
	/	/	E-232 & R-119	2.9
	/	/	I-233 & C-128	3.0
	/	/	I-233 & H-124	3.4
	/	/	I-233 & M-126	3.3
	/	/	F-234 & C-121	3
	/	/	F-234 & R-120	3.7
	/	/	I-233 & C-128	3
	/	/	Q-235 & H-124	3.1
	/	/	Q-235 & C-127	2.7
	/	/	R-236 & A-125	3.3
	/	/	Q-237 & A-125	3.4

Texts in red indicate the strongly effective interaction sites with inner distance less than 3 Å.

### Conclusions and Perspectives

**Figure 5:**
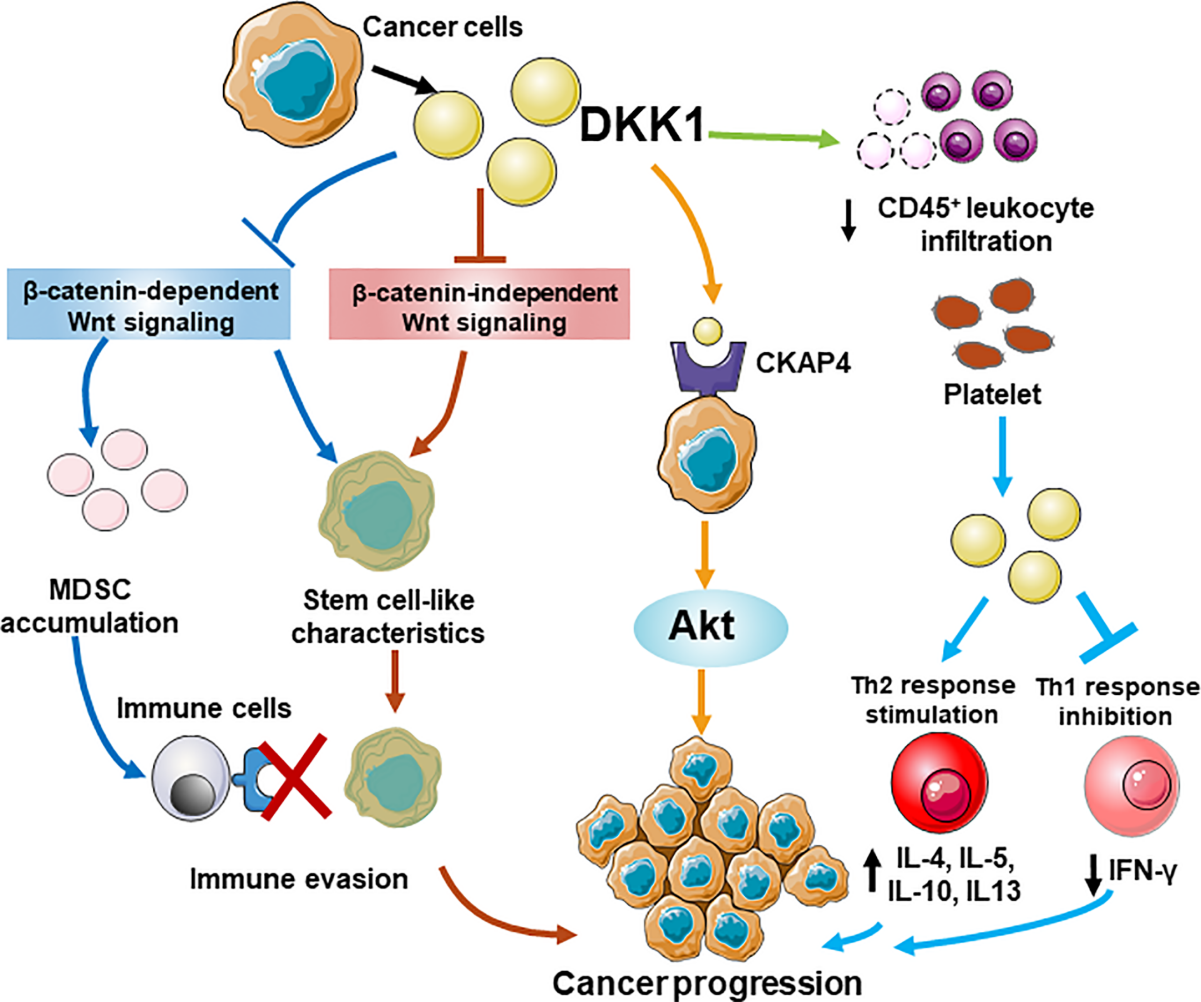
A schematic model for different roles of DKK1 in immunomodulatory and tumorigenesis. DKK1 promotes an immunosuppressive tumor microenvironment that benefited MDSCs expansion by regulating β-catenin-independent Wnt signaling, and suppressing the proliferation of CD45^+^ T cells, thus contributing to cancer immune evasion. DKK1 helps cancer cells possess stem cell-like properties by preventing Wnt activation regardless of β-catenin dependence. A novel pathway shows DKK may interact with CKAP4 receptor, which lead to the activation of Akt signaling and increases the proliferation of the cancer cells. In the allergen challenge model, Platelet-derived DKK1 enhances Th2 response and elevates the secretion of IL-4, IL-5, IL-10, and IL-13, while suppresses Th1 response by reducing IFn-γ expression.

Overall, DKK1 is an ideal anti-cancer therapeutic target for immunotherapy. Despite its role as clinical biomarker of cancer progression and prognosis, it also participated in multiple tumor deterioration in preclinical cancer models. The elevated serum level of DKK1 was observed in patients of various cancers with poor prognosis. According to gene profile analysis, mRNA level of *DKK1* was overexpressed in a range of tumor types (14 out of 27). In addition to mRNA level, DKK1 protein level was found to be increased in both serum and in tissue samples in some specific cancers. There was a positive correlation between the mRNA level of *CTNNB1* and *DKK1* according to our calculation, supported by the similar evidence from protein expressions in several types of cancers, suggesting that the elevation of DKK1 may be attributed to overactivation of Wnt signaling. More studies are warranted to unravel the mechanism of DKK1 protein secretion and its existence in cancer. Preclinically, DKK1 was proven to promote proliferation, metastasis, and invasion in cancer cell lines and DKK1 inhibition showed efficacy on tumor regression in animal models. Furthermore, DKK1 demonstrated immunoregulatory impacts including encouraging Th1/Th2 shifts, promote an immunosuppressive tumor microenvironment that benefited MDSCs expansion and T cell suppression, reducing the functionality of T cells by MDSC modulation, and suppressing the proliferation of CD8^+^ T cells and NK cells, thus contributing to inflammatory response and cancer immune evasion. Although the functional mechanism of DKK1 has not yet been fully elucidated, its role in signaling pathways in cancers and immune cells has become increasingly apparent. The engaging pathways include the canonical β-catenin-dependent Wnt signaling, β-catenin-independent Wnt signaling and the novel one involving CKAP4. Extra work is required to understand coordination of DKK1 in these pathways. A schematic model of different roles of DKK1 in immunomodulatory and tumorigenesis is illustrated (**Figure 5**). The latest findings from clinical trials of anti-DKK1 antibody DKN-01 also demonstrated that DKK1 is a potential candidate for treating advanced GEJ/GC and gynecologic cancers. The therapeutic strategy of developing a combination of DKK1 and immune checkpoint inhibitor based on precision medicine is promising for tumor patients with high DKK1 expression. As mentioned, DKK1 was highly expressed in a great variety of cancers and also involved in cancer progression. For instance, DKK1 was involved in bone metastases of small cell lung cancer cell model (58). It is reasonable to speculate that DKK1 could be a therapeutic approach for a wide range of malignancies. Based on these results and the increasing evidence of DKK1 tumor promoting activity, further clinical development is warranted. Another intriguing point in the clinical results is that in the combination treatment with DKN-1 and pembrolizumab, patients with a high DKK1 level in tumor demonstrated relatively higher efficacy than those with a low level. The same effect was observed in patients who were resistant to PD-1 treatment. Moreover, DKN-01 monotherapy had much less efficacy than that of combination therapy with anti-PD-1 antibody (51). With the aim to improve the performance of DKK1 monotherapy, it is imperative to elucidate the structural mechanism of DKK1 inhibition of the Wnt pathway. As mentioned, the N domain of DKK1 may be eligible to regulate the ability of its own C domain binding to LRP6. Our computational analysis results were consistent with this and pointed out the possibility of two distinct conformations of DKK1 structure with different interaction of N and C domain. Based on these points, we can study how different configurations of N-terminal domains of DKK1 affect the Wnt signaling pathway through interaction with the C-terminal domains thereby discovering the new targeting domain on DKK1 for further drug development.

## Author Contributions

HC wrote and revised the manuscript. ZC performed the data analysis for DKK1 expression in cancers and the DKK1 structure prediction. HC, LW, Z-KZ, XT, SL, B-TZ, and AL contributed the manuscript for literature research. YY and GZ revised and approved the manuscript. All authors contributed to the article and approved the submitted version.

## Funding

This study was supported by Hong Kong General Research Fund from the Research Grants Council of the Hong Kong Special Administrative Region, China (Project No. 12102120), Theme-based Research Scheme from the Research Grants Council of the Hong Kong Special Administrative Region, China (Project No. T12-201/20-R), Basic and Applied Basic Research Fund from Department of Science and Technology of Guangdong Province (Project No. 2019B1515120089), Inter-institutional Collaborative Research Scheme from Hong Kong Baptist University (Project No. RC-ICRS/19-20/01) and University-Industry Collaboration Programme from Innovation and Technology Commissions of the Hong Kong Special Administrative Region, China (Project No. UIM/328).

## Conflict of Interest

The authors declare that the research was conducted in the absence of any commercial or financial relationships that could be construed as a potential conflict of interest.
